# Vitamin C deficiency reveals developmental differences between neonatal and adult hematopoiesis

**DOI:** 10.3389/fimmu.2022.898827

**Published:** 2022-09-30

**Authors:** Ira Phadke, Marie Pouzolles, Alice Machado, Josquin Moraly, Pedro Gonzalez-Menendez, Valérie S. Zimmermann, Sandrina Kinet, Mark Levine, Pierre-Christian Violet, Naomi Taylor

**Affiliations:** ^1^ Pediatric Oncology Branch, Center for Cancer Research, National Cancer Institute, National Institutes of Health (NIH), Bethesda, MD, United States; ^2^ Institut de Génétique Moléculaire de Montpellier, University of Montpellier, Centre National de la Recherche Scientifique (CNRS), Montpellier, France; ^3^ Laboratory of Excellence GR-Ex, Paris, France; ^4^ Molecular and Clinical Nutrition Section, Intramural Research Program, National Institute of Diabetes and Digestive and Kidney Diseases, National Institutes of Health, Bethesda, MD, United States

**Keywords:** vitamin C, ascorbate, GULO, hematopoiesis, erythropoiesis, anemia, neonatal, development

## Abstract

Hematopoiesis, a process that results in the differentiation of all blood lineages, is essential throughout life. The production of 1x10^12^ blood cells per day, including 200x10^9^ erythrocytes, is highly dependent on nutrient consumption. Notably though, the relative requirements for micronutrients during the perinatal period, a critical developmental window for immune cell and erythrocyte differentiation, have not been extensively studied. More specifically, the impact of the vitamin C/ascorbate micronutrient on perinatal as compared to adult hematopoiesis has been difficult to assess in animal models. Even though humans cannot synthesize ascorbate, due to a pseudogenization of the L-gulono-γ-lactone oxidase (*GULO*) gene, its generation from glucose is an ancestral mammalian trait. Taking advantage of a *Gulo^-/-^* mouse model, we show that ascorbic acid deficiency profoundly impacts perinatal hematopoiesis, resulting in a hypocellular bone marrow (BM) with a significant reduction in hematopoietic stem cells, multipotent progenitors, and hematopoietic progenitors. Furthermore, myeloid progenitors exhibited differential sensitivity to vitamin C levels; common myeloid progenitors and megakaryocyte-erythrocyte progenitors were markedly reduced in *Gulo^-/-^* pups following vitamin C depletion in the dams, whereas granulocyte-myeloid progenitors were spared, and their frequency was even augmented. Notably, hematopoietic cell subsets were rescued by vitamin C repletion. Consistent with these data, peripheral myeloid cells were maintained in ascorbate-deficient *Gulo^-/-^* pups while other lineage-committed hematopoietic cells were decreased. A reduction in B cell numbers was associated with a significantly reduced humoral immune response in ascorbate-depleted *Gulo^-/-^* pups but not adult mice. Erythropoiesis was particularly sensitive to vitamin C deprivation during both the perinatal and adult periods, with ascorbate-deficient *Gulo^-/-^* pups as well as adult mice exhibiting compensatory splenic differentiation. Furthermore, in the pathological context of hemolytic anemia, vitamin C-deficient adult *Gulo^-/-^* mice were not able to sufficiently increase their erythropoietic activity, resulting in a sustained anemia. Thus, vitamin C plays a pivotal role in the maintenance and differentiation of hematopoietic progenitors during the neonatal period and is required throughout life to sustain erythroid differentiation under stress conditions.

## Introduction

Vitamin C (ascorbic acid, ascorbate), is an essential micronutrient to humans, possessing antioxidant activities. Vitamin C has been described as a regulator of numerous physiological processes including neuronal development (1), collagen synthesis (2), immune cell differentiation and function (3), response to infection (4), and most recently, in anti-cancer therapies (5, 6). Mechanistically, vitamin C serves as a cofactor for Jumonji-C domain-containing histone demethylases (JHDMs) and the ten-eleven translocation (TET) dioxygenases (7), altering the epigenome of the cell (7). An abnormal epigenetic remodeling leads to a dysregulated differentiation of stem cells, leukemic stem cells, as well as lymphocytes, amongst others (8–15). Moreover, vitamin C can impact cell physiology by scavenging reactive oxygen species (16). In the context of erythroid differentiation, oxidized ascorbate increases mitochondrial superoxide and exacerbates abnormal erythroblast differentiation whereas vitamin C, scavenging reactive oxygen species and reprogramming mitochondrial metabolism, rescues erythropoiesis (17). Thus, vitamin C regulates a myriad of cell processes through a wide diversity of mechanisms.

Of the >4,000 species of mammals, only higher primates, guinea pigs, and fruit bats have lost the ability to synthesize vitamin C from glucose, due to inactivation of L-gulono-γ-lactone oxidase (GULO), the enzyme that catalyzes the terminal step of L-ascorbic acid biosynthesis (18). Humans and guinea pigs independently lost the ability to synthesize ascorbate 40-50 and 20-25 million years ago, respectively, and the loss in bats was more recent (19–22). These data, together with the strong pseudogenization of the *Gulo* gene in higher primate and guinea pig genomes, strongly suggest that there was a selective pressure against vitamin C synthesis (22). From a dietary perspective, this pseudogenization has resulted in a requirement that these species consume diets that are rich in vitamin C, with severe vitamin C deficiency resulting in scurvy (16, 22). Nevertheless, prior to the dramatic onset of scurvy, vitamin C deficiency can negatively impact a myriad of other cellular processes. Based on our recent finding that vitamin C accelerates *ex vivo* human erythroid differentiation (17), it is of much interest to evaluate the impact of vitamin C levels on *in vivo* hematopoiesis, with a specific focus on erythroid differentiation.

To elucidate the role of vitamin C in regulating physiological *in vivo* hematopoiesis, we took advantage of the *Gulo^-/-^* mouse model which is dependent on dietary vitamin C for long-term survival (23, 24). Furthermore, as hematopoiesis is a dynamic process that changes as a function of ontogeny, we evaluated this process during the immediate postnatal period as well as in adults. During mouse development, hematopoiesis starts in the yolk sac and aorta-gonad-mesonephros region post conception, then moves to the placenta, fetal liver and spleen, before being sustained in the bone marrow (BM) from day 17.5 dpc (days post coitum) throughout the life of the animal (25–28). There are significant differences between fetal liver compared to postnatal BM HSCs, with the former showing a greater repopulating capacity than BM-HSCs (29–31). Moreover, BM hematopoietic stem cells (HSCs) from juvenile mice (<3-4 weeks of age) exhibit a much higher proliferative capacity than HSCs from adult mice (32). Notably though, differences in the metabolic regulation of BM HSCs in neonates and adults have not been extensively evaluated. Here, we find that ascorbic acid deficiency has a significantly greater impact on multiple hematopoietic differentiation pathways during the immediate postnatal period than in adults. Dramatic decreases in HSCs as well as multipotent progenitors (MPPs) and hematopoietic progenitors (HPCs) were associated with reduced numbers of common myeloid progenitors (CMPs) and megakaryocyte-erythrocyte progenitors (MEPs). Our data also show important differences in the sensitivity of progenitors to ascorbate levels–GMPs and mature myeloid cells were not affected while B and erythroid lineage cells were markedly reduced. Furthermore, while anemia resulting from vitamin C deprivation during the adult period was associated with a compensatory splenic erythropoiesis, these mice were unable to mount an efficient response to a hemolytic anemia. Thus, our data unveil a crucial role for vitamin C metabolism in supporting hematopoiesis during the postnatal period and highlight the importance of ascorbate in promoting erythropoiesis under stress conditions.

## Results

### Ascorbate availability regulates the fate of BM hematopoietic progenitor populations in neonatal and adult GULO^-/-^ mice

**Figure 1:**
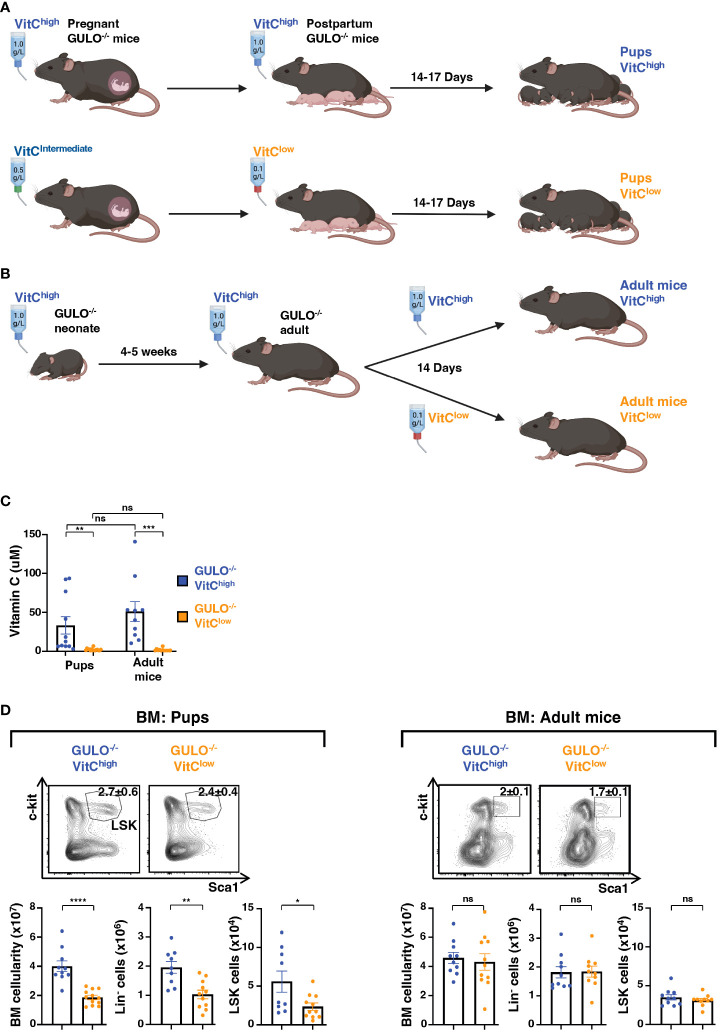
Ascorbate deficiency in neonatal GULO^-/-^ mice results in a significant reduction in bone marrow hematopoietic progenitors. **(A)** Schema illustrating the protocol used to generate ascorbate-deficient GULO^-/-^ pups. GULO^-/-^ pregnant dams were supplemented with vitamin C (VitC) in the drinking water, either at 1g/L for the high dose group (upper panel) and 0.5g/L for the intermediate dose group (lower panel). Following the birth of pups, dams were either maintained at the high dose of vitamin C (1g/L) or the dose was decreased to 0.1g/L in the low dose group. **(B)** Schema illustrating the protocol used to generate adult GULO^-/-^ mice (6-7 weeks) with ascorbate deficiency, by decreasing vitamin C in the drinking water of adult mice (4-5 weeks of age) from 1.0g/L to 0.1g/L for 14 days. **(C)** Plasma vitamin C concentrations were measured by HPLC in pups and adult mice maintained on high or low dose vitamin C as detailed in panels **(A, B)** Means ± SEM are presented (n=10-15 mice per group). **(D)** Total bone marrow (BM) cells, lineage^-^ hematopoietic progenitors (Lin^-^), and Lin^-^Sca1^+^c-kit^+^ (LSK) progenitors were quantified in pups (left) and adult (right) GULO^-/-^ mice supplemented with 1g/L of vitamin C (GULO^-/-^VitC^high^, blue) or 0.1g/L of vitamin C (GULO^-/-^VitC^low^, orange). Representative contour plots showing the c-kit/Sca1 profiles in Lin^-^ cells are presented and means ± SEM are indicated (n=9-12 mice per group). Statistical differences were evaluated by an unpaired two-tailed t-test. *p<0.05; **p < 0.01; ***p < 0.001; ****p < 0.0001; ns, not significant.

Here, we studied the impact of ascorbate deficiency on hematopoiesis, evaluating potential differences during the neonatal and adult periods. To deplete mice of ascorbate during the neonatal period, pregnant *Gulo^−/−^* dams were first supplemented with an intermediate dose of vitamin C (0.5g/L) in their drinking water as compared to the standard high dose of 1.0g/L, and then immediately postpartum, vitamin C was decreased to 0.1g/L in the lactating dams (**Figure 1A**). The lowest vitamin C dose of 0.1g/L was not used during pregnancy as this level of deprivation resulted in a high mortality of pups. To evaluate the impact of ascorbate deficiency in the adult period, *Gulo^−/−^* mice were maintained in high dose ascorbate until 3-4 weeks of age and then deprived of vitamin C (supplementation decreased to 0.1g/L) for 14 days (**Figure 1B**). At the high dose of of vitamin C, plasma vitamin C levels were similar to those reported in WT mice (**Figure 1C**), ranging from 50-90μM (33, 34). The 2 week time period was selected because vitamin C levels in plasma and organs are significantly decreased by 1 week after withdrawal (34, 35), allowing evaluation of hematopoiesis during the neonatal period. Indeed, following vitamin C deprivation, ascorbate levels dropped markedly, from 33±11 and 51±13 μM in *Gulo^−/−^* pups and adults, to 2.2±0.4 and 1.9±0.5 μM, respectively (p<0.01, **Figure 1C**). Notably, while ascorbate levels were lower in *Gulo^−/−^* pups than adult mice receiving high doses of vitamin C, ascorbate deficiency during the neonatal period resulted in a generalized reduction in bone marrow cellularity in neonatal *Gulo^−/−^* mice, decreasing from 40.1x10^6^±3.7x10^6^ to 18.6x10^6^±1.5x10^6^ (p<0.0001) and this reduction was associated with a >2-fold reduction in both lineage-negative (Lin^-^) and Lin^-^Sca1^+^c-kit^+^ (LSK) hematopoietic progenitors (p<0.05, **Figure 1D**). This was not the case in ascorbate-deprived adult *Gulo^−/−^* mice where neither BM cellularity nor the numbers of LSK progenitors was altered (**Figure 1D**). These data suggested that ascorbate depletion has a more profound impact on the fate of BM progenitors in neonatal than adult mice.

**Figure 2:**
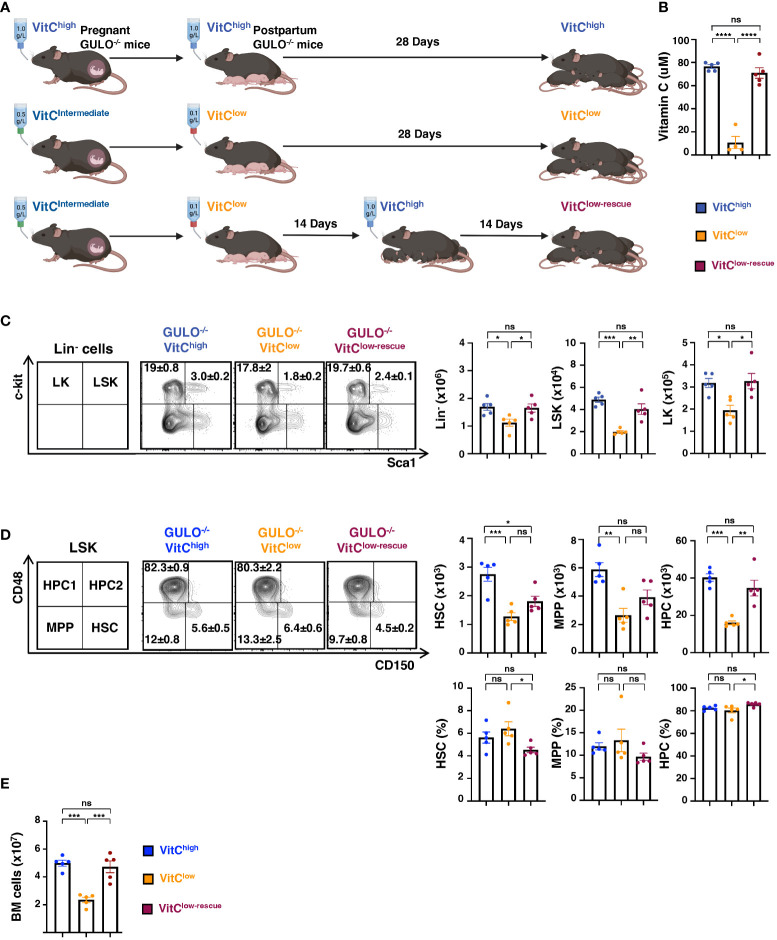
Ascorbate rescue results in the increased generation of BM hematopoietic progenitors in GULO^-/-^ pups. **(A)** Schema illustrating the rescue of ascorbate-deficient neonatal GULO^-/-^ mice. Following the birth of pups, dams were either maintained at a high (1g/L, top) or low (0.1g/L, middle) dose of vitamin C for 28 days. For rescue experiments, GULO^-/-^ pups maintained on low dose VitC (0.1g/L) were switched to a high dose (1g/L) at 14 days of life and mice were evaluated at day 28 (VitC^low-rescue^, bottom). **(B)** Plasma VitC concentrations were measured in all 3 groups at day 28 and means ± SEM are presented (n=4-5 per group). **(C)** Representative c-Kit/Sca1 plots of Lin^-^ cells are shown (left) and absolute numbers ± SEM of Lin^-^, LSK, and LK progenitors are presented (n=5 mice per group). **(D)** Gating strategy used to define HSC (CD150^+^CD48^-^LSK), MPP (CD150^-^CD48^-^LSK) and HPC (CD150^+/-^CD48^+^LSK) populations (36) together with quantifications and percentages of each subset ± SEM are presented (n=5 mice per group). **(E)** Mean BM cellularity ± SEM in each condition (n=5 per group). Statistical analyses were performed using a one-way ANOVA (Tukey’s test). *p < 0.05; **p < 0.01; ***p < 0.001; ****p < 0.0001; ns, not significant.

To determine whether the decreased BM cellularity in *Gulo^−/−^* neonatal mice was specifically due to ascorbate depletion, 14 day old ascorbate-deficient *Gulo^−/−^* pups were either maintained on low vitamin C supplementation or switched to high vitamin C supplementation for an additional 14 days (*Gulo^−/−^* VitC^low-rescue^, **Figure 2A**). The 14 day rescue period was sufficient to restore ascorbate levels to baseline, increasing from 11±5 to 71±5 μM (p<0.0001, **Figure 2B**) and notably, Lin^-^, LSK, and LK (Lin^-^Sca1^-^c-kit^+^) progenitors were all significantly augmented, to levels similar to those detected in *Gulo^−/−^* VitC^high^ mice (**Figure 2C**). As the CD150 and CD48 SLAM markers can subdivide LSK into HSCs with long term reconstitution activity (CD150^+^CD48^−^LSK), multipotent progenitors (MPPs, CD150^-^CD48^−^LSK) and heterogeneous restricted progenitors (HPCs, CD150^-/+^CD48^+^LSK) (36–38), we evaluated their profiles in *Gulo^−/−^* VitC^high^, *Gulo^−/−^* VitC^low^, and *Gulo^−/−^* VitC^low-rescue^ mice. Importantly, the numbers of BM HSCs, MPPs, and HPCs were all significantly reduced in *Gulo^−/−^* VitC^low^ mice as compared to *Gulo^−/−^* VitC^high^ mice (p<0.01, **Figure 2D**). Ascorbate rescue resulted in a trend towards increased HSCs and MPPs, and HPCs were markedly augmented, increasing from 16.0x10^3^±1.0x10^3^ to 34.6x10^3^±4.2x10^3^ (p<0.01, **Figure 2D**). This enhanced level of hematopoiesis correlated with a decreased percentage of BM HSC as well as an increased percentage of HPC following ascorbate rescue (p<0.05, **Figure 2D**). These data, together with an impressive increase in BM cellularity following vitamin C rescue, from 2.3x10^7^±0.2x10^7^ to 4.7x10^7^±0.4x10^7^ (p<0.001, **Figure 2E**), strongly suggest that adequate vitamin C levels are a prerequisite for HSC differentiation and the expansion of lineage committed hematopoietic cells.

### Myeloid cell progenitors are differentially impacted by ascorbate deficiency

**Figure 3:**
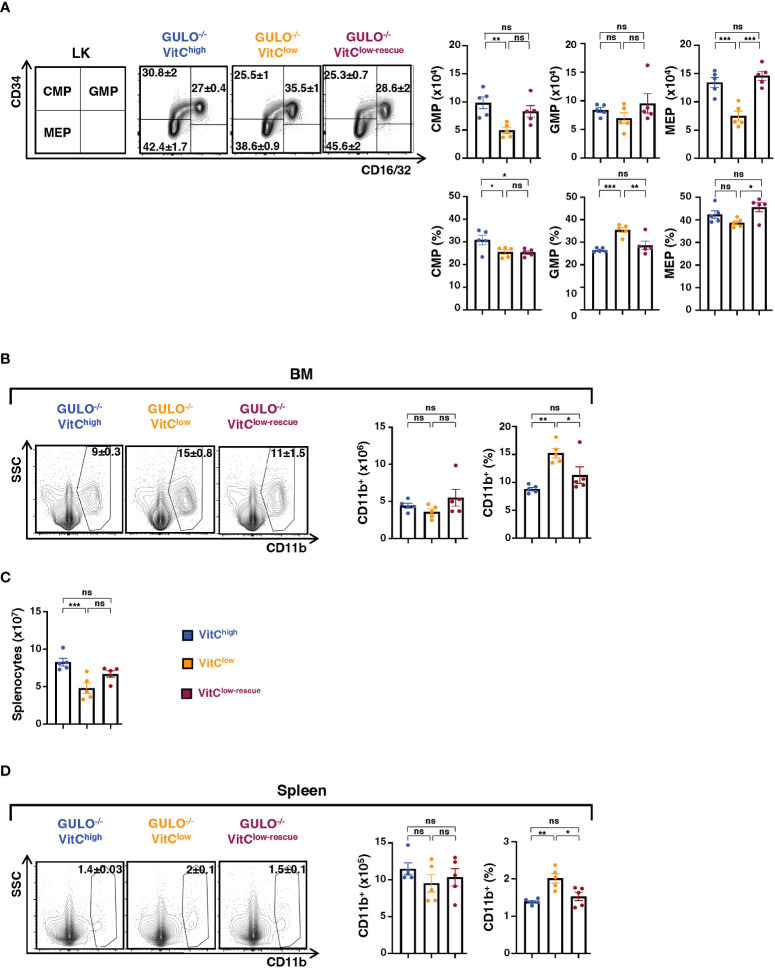
Differential impact of ascorbate deprivation on myeloid progenitor subsets. (**A** Gating strategy used to define CMP (CD34^+^CD16/32^-^LK), GMP (CD34^+^CD16/32^+^LK), and MEP (CD34^-^CD16/32^-^LK) subsets are shown together with representative contour plots. Quantifications and percentages of each subset ± SEM in 28 day old GULO^-/-^ mice maintained under VitC-high, VitC-low, and rescue conditions are presented (n=5 mice per group). **(B)** CD11b^+^ myeloid cells were evaluated in BM of the indicated groups by flow cytometry and representative contour plots are shown (left). Total numbers as well as percentages of CD11b+ cells are presented as means ± SEM (n=5 mice per group). **(C)** Splenic cellularity ± SEM in each condition is shown (n=5 mice per group). **(D)** CD11b^+^ myeloid cells in spleen were evaluated as in panel **(B)**. Means ± SEM are shown (n=5 mice per group). Statistical differences were analyzed using a one-way ANOVA (Tukey’s test). *p < 0.05; **p < 0.01; ***p < 0.001; ns, not significant.

HSCs undergo commitment to multipotent common myeloid progenitors (CMPs, CD34^+^CD16/32^-^LK) cells which can then differentiate into granulocyte-monocyte progenitors (GMPs, CD34^+^CD16/32^+^LK) or megakaryocyte-erythrocyte progenitors (MEPs, CD34^-^CD16/32^-^LK) (39–41). In accord with the HPC data (**Figure 2E**), the numbers of CMPs were reduced in ascorbate-deficient mice and increased to baseline following ascorbate rescue (**Figure 3A**). Interestingly, the total numbers of GMPs were not decreased in ascorbate-deficient conditions and indeed, GMP percentages were actually increased (p<0.001, **Figure 3A**). In contrast, MEPs were extremely sensitive to ascorbate levels, with a significantly lower level of MEP in *Gulo^−/−^* VitC^low^ mice, and a rescue in these levels following ascorbate supplementation (p<0.001, **Figure 3A**). These results reveal differences in the sensitivity of myeloid progenitors to ascorbate levels. Furthermore, the finding that mature CD11b^+^ myeloid cell numbers were similar in *Gulo^−/−^* VitC^high^, *Gulo^−/−^* VitC^low^, and *Gulo^−/−^* VitC^low-rescue^ mice suggests that this lineage is not markedly impacted by BM ascorbate concentrations (**Figure 3B**). These data are consistent with a significantly higher frequency of CD11b^+^ BM myeloid cells under conditions of ascorbate deficiency and a decreased frequency upon ascorbate rescue (**Figure 3B**).

The decrease in BM hematopoietic progenitors was coupled to a significantly reduced splenic cellularity in *Gulo^−/−^* VitC^low^ as compared to *Gulo^−/−^* VitC^high^ mice, and numbers trended upwards following rescue (**Figure 3C**). However, consistent with the lack of impact of ascorbate deficiency on BM myeloid cells, the numbers of CD11b^+^ splenocytes were not altered by either ascorbate deprivation or rescue and again, the frequency of CD11b^+^ splenocytes was increased in ascorbate-deprived *Gulo^−/−^* mice (p<0.01, **Figure 3D**). The ensemble of these data reveals the importance of ascorbate-coupled regulation in the fate of early BM hematopoietic progenitors. Additionally, given the short half-life of myeloid cells (42), our data indicate that myeloid cell differentiation continues under conditions of ascorbate acid deficiency.

### Ascorbate deficiency results in defective B lymphocyte differentiation with attenuated immunoglobulin production during the neonatal period

The hypocellular BM and spleen in ascorbate-deficient *Gulo^−/−^* mice (**Figures 2E**, **3C**), together with a significant increase in the frequency of myeloid cells (**Figures 3B, D**), suggested that ascorbate levels might impact lymphoid lineage differentiation. We therefore evaluated both T and B lymphocyte differentiation in *Gulo^−/−^* mice as a function of their ascorbate status. Interestingly, *ex vivo* T cell differentiation (43) as well as the differentiation of regulatory and IL-17-secreting T cell subsets have been shown to be sensitive to ascorbic acid levels, *via* the vitamin C-regulated activity of Ten-eleven translocation (TET) enzyme dioxygenases (8, 9, 12, 14, 15). Importantly though, thymocyte differentiation in 14-day old *Gulo^−/−^* pups was globally intact, with similar absolute numbers of thymocytes as well as the different thymocyte sub-populations; double negative (DN), double positive (DP), and more mature single positive CD4 (SP4), single positive CD8 (SP8), and γδ thymocyte subsets (**Figures S1A, B**). Only the numbers of CD8^+^ intermediate single positive (ISP) thymocytes, representing a stage of TCR β-selection rearrangement wherein there is a proliferative burst (44–48), were lower in ascorbate-deficient as compared to ascorbate-sufficient *Gulo^−/−^* pups (**Figure S1B**). Indeed, in agreement with previous literature (10, 34), the numbers of splenic CD4^+^ and CD8^+^ T cells (**Figure S1C**) as well as subsets of naïve, central memory and effector T cells (data not shown) were similar in *Gulo^−/−^* VitC^low^ and *Gulo^−/−^* VitC^high^ pups as well as adult mice. However, an extended 28 day ascorbate deprivation starting at birth led to a significant decrease in the total number of splenic CD3^+^ T cells (**Figure S1D**). Moreover, it is interesting to note that this did not reflect a global decrease in T lymphocytes as CD8^+^, but not CD4^+^, T cells were significantly reduced following ascorbate deprivation and were rescued by ascorbate supplementation (**Figure S1D**). Thus, long-term ascorbate deprivation during the neonatal period negatively impacts peripheral CD8^+^ T cell maintenance and/or homeostatic proliferation.

**Figure 4:**
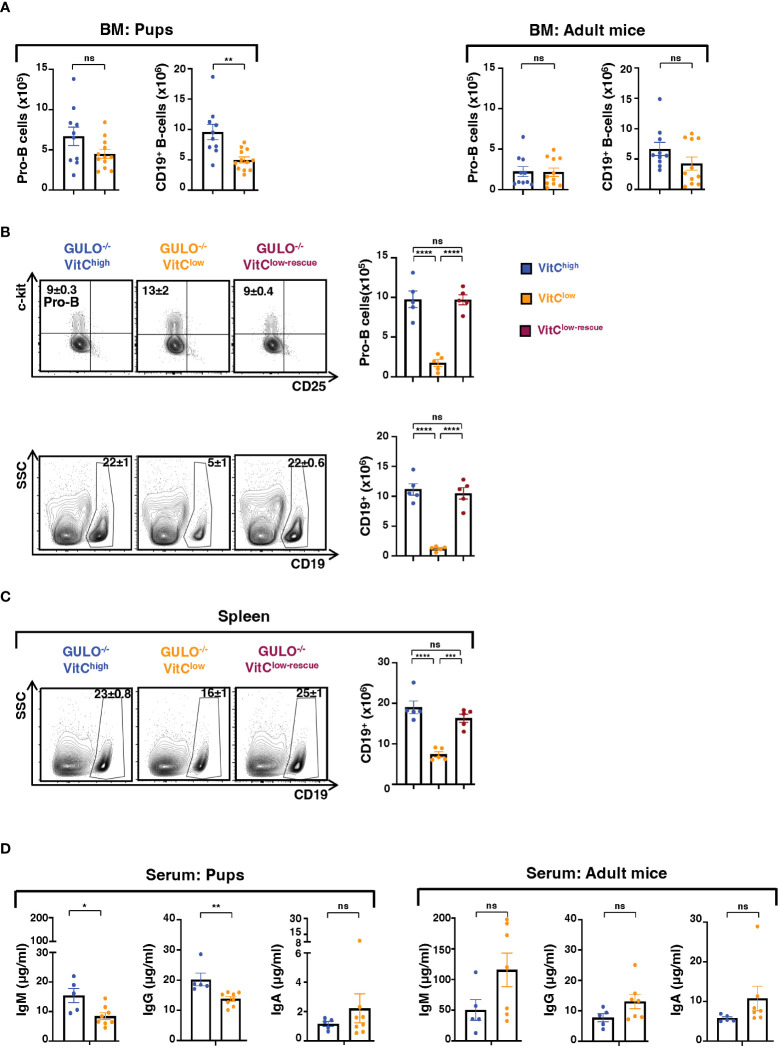
Ascorbate deprivation during the neonatal period results in a significant loss in B cell lymphopoiesis and reduced immunoglobulin production. **(A)** B cell differentiation was evaluated in the BM of pups (left) and adult (right) GULO^-/-^ mice supplemented with 1g/L of vitamin C (GULO^-/-^VitC^high^) or 0.1g/L of vitamin C (GULO^-/-^VitC^low^). Total numbers of pro-B cells, evaluated as CD19^+^c-kit^+^CD25^-^, and CD19^+^ B cells were quantified and means ± SEM are shown (n=10-12 mice per group). **(B)** Pro-B and B lymphocytes were evaluated at day 28 in BM of GULO^-/-^VitC^high^, GULO^-/-^VitC^low^, and GULO^-/-^VitC^low-rescue^ mice. Representative contour plots as well as absolute numbers of pro-B and B cells are shown (n=5 mice per group). **(C)** Representative contour plots of CD19^+^ splenic B cells in 28 day old GULO^-/-^ mice maintained under VitC-high, VitC-low, and rescue conditions together with absolute numbers of splenic B cells (n=5 mice per group). **(D)** Serum immunoglobulins (IgM, IgG and IgA) were monitored in neonatal (18 day old, left) and adult (right) GULO^-/-^ mice supplemented with high or low vitamin C by ELISA. Means ± SEM (μg/ml) are presented (n= 5-8 mice per group). Statistical analyses were performed using an unpaired two-tailed t-test in panels **(A, D)**; and a one-way ANOVA (Tukey’s test) in panels B-C. *p < 0.05; **p < 0.01; ***p < 0.001; ****p < 0.001; ns, not significant.

B lymphocyte differentiation during the neonatal period was acutely sensitive to vitamin C levels as CD19^+^ BM B cells were significantly reduced by a 14-17 day ascorbate deprivation (p<0.01, **Figure 4A**). However, BM B cells in adult *Gulo^−/−^* mice were not impacted by vitamin C depletion and splenic B cell numbers remained high (**Figures 4A** and **S2**). While the numbers of c-kit^+^ pro-B progenitors in ascorbate-deprived neonatal mice trended lower in *Gulo^−/−^* VitC^low^ than *Gulo^−/−^* VitC^high^ mice, these levels did not reach significance (**Figure 4A**). Notably though, an extended 28 day vitamin C deprivation period during the neonatal period resulted in a massive loss in pro-B cells in the BM, decreasing from 9.8x10^5^±1.1x10^5^ to 1.7x10^5^±0.4x10^5^ (p<0.0001, **Figure 4B**), and this was associated with an almost complete loss in CD19+ B cells (11.2x10^6^±1.0x10^6^ to 1.2x10^6^±0.2x10^6^, p<0.0001 **Figure 4B**). The reduction in B cell differentiation was directly due to low ascorbate levels as a 14 day rescue with high vitamin C supplementation restored pro-B as well as mature B cells in the BM (**Figure 4B**). Furthermore, the reduction in splenic B cells was alleviated by vitamin C supplementation (**Figure 4C**). Thus, ascorbate deficiency results in a dramatic decrease in B cell differentiation.

The important impact of vitamin C supplementation on B cell differentiation during the neonatal period raised the possibility that immunoglobulin production might be impacted by plasma/BM ascorbate levels. Indeed, basal IgM and IgG levels were significantly lower in ascorbate-deficient as compared to ascorbate-sufficient *Gulo^−/−^* pups (p<0.05-0.01, **Figure 4D**) whereas they were not altered in *Gulo^−/−^* adult mice (**Figure 4D**). Ascorbic acid supplementation has been shown to positively regulate plasma cell differentiation in adult *Gulo^−/−^* mice (49) and the data presented here add to our understanding of vitamin C metabolism in B lymphocytes. During the first two weeks of postnatal life, a period that is characterized by an impaired ability to mount a humoral response (50), ascorbic acid levels play a critical role in B cell function, regulating immunoglobulin production.

### BM and splenic erythroid lineage cells are negatively impacted by ascorbic acid deprivation during the neonatal period

**Figure 5:**
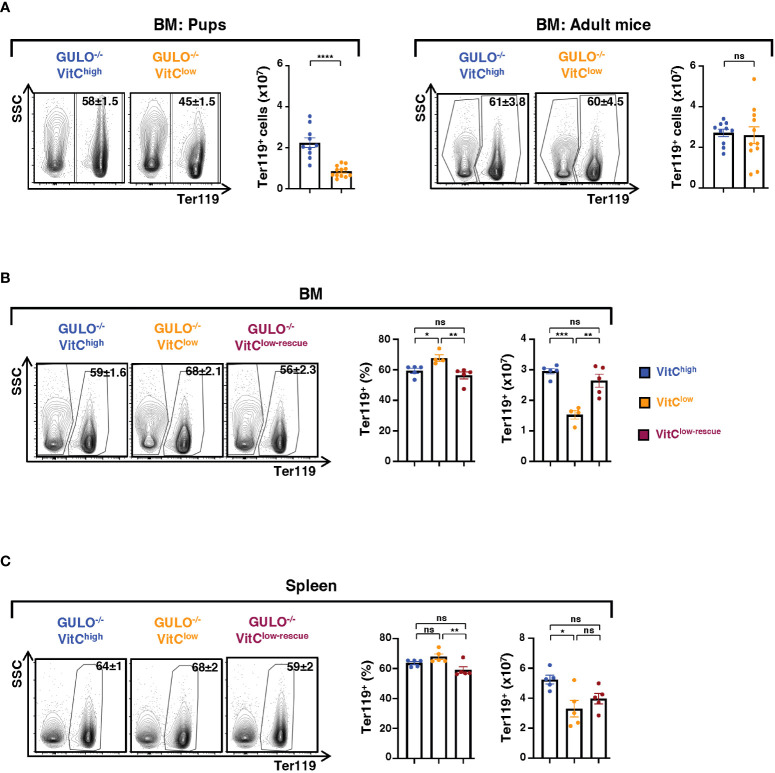
Ascorbate deprivation leads to decreased erythroid cells in GULO^-/-^ pups. **(A)** Ter119^+^ erythroid BM cells were quantified in pups (left) and adult (right) GULO^-/-^ mice supplemented with 1g/L of vitamin C (GULO^-/-^VitC^high^) or 0.1g/L of vitamin C (GULO^-/-^VitC^low^; n=10-12 mice per group). Representative contour plots are shown and quantification of Ter119^+^ cells are presented as means ± SEM. **(B)** Ter119^+^ BM cells were evaluated in 28 day old GULO^-/-^ mice maintained under VitC-high, VitC-low, and rescue conditions. The percentages and absolute numbers of Ter119^+^ cells are shown (n=5 per group). **(C)** Splenic Ter119^+^ erythroid cells were evaluated as in panel **(B)** and means ± SEM are presented (n=5 mice per group). Statistical differences were analyzed using an unpaired two-tailed t-test in panel **(A)**; and a one-way ANOVA (Tukey’s test) in panels **(B, C)**. *p < 0.05; **p < 0.01; ***p < 0.001; ****p < 0.0001; ns, not significant.

Based on the hypocellularity of the BM of ascorbate-deficient neonatal *Gulo^−/−^* mice and the dramatic decrease in MEPs (**Figure 3A**), we quantified Ter119^+^ erythroid cells–a lineage that comprises a large proportion of BM hematopoietic cells (51). In agreement with the BM hypocellularity in ascorbate-deficient *Gulo^−/−^* pups, the absolute number of Ter119^+^ BM cells was significantly reduced following ascorbate deprivation; 8.4x10^6^±0.8x10^6^ and 22.4x10^6^±2.4x10^6^ erythroid cells in ascorbate-deficient and sufficient pups, respectively (p<0.001, **Figure 5A**). In contrast, the numbers of Ter119^+^BM cells in adult *Gulo^−/−^* VitC^low^ mice revealed no significant differences with *Gulo^−/−^* VitC^high^ mice (**Figure 5A**), in accord with their normal BM cellularity (**Figure 1D**). Finally, vitamin C supplementation at 14 days of life resulted in a complete restoration of Ter119^+^ BM cell numbers by day 28 (p<0.01, **Figure 5B**). Interestingly though, the percentages of Ter119^+^ erythroid cells in the spleen were not significantly augmented, suggesting differences in the regulation of erythroid differentiation in these organs (**Figure 5C**).

**Figure 6:**
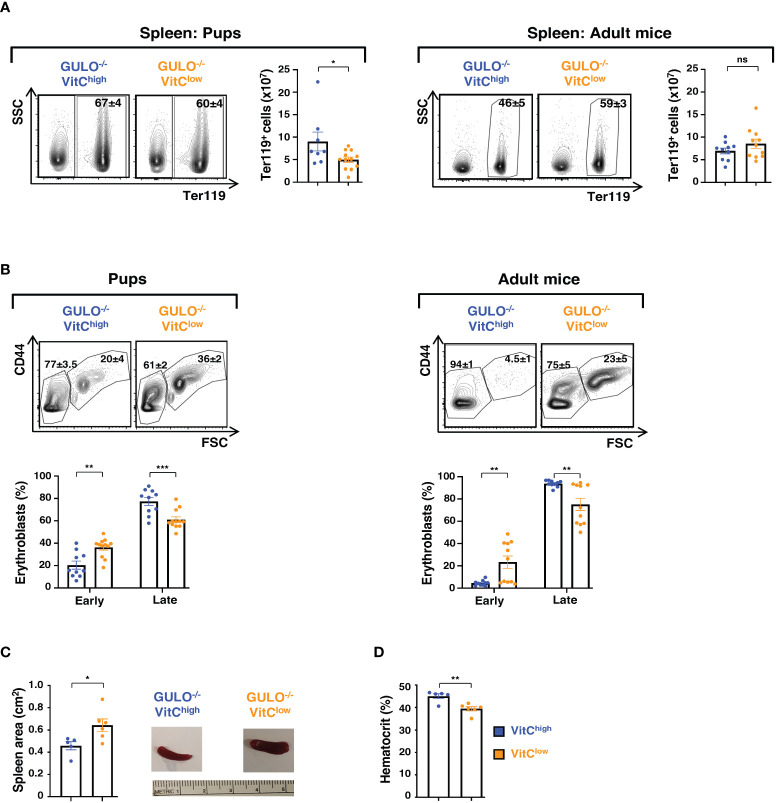
Ascorbate deficiency is associated with splenic erythropoiesis in GULO^-/-^ mice. **(A)** Ter119^+^ cells in the spleen were assessed in GULO^-/-^ pups and adult mice in the indicated conditions. Representative contour plots and quantifications are presented. **(B)** Within the splenic Ter119^+^ subset, early (pro, basophilic and polychromatic) erythroblasts were distinguished from late (orthochromatic) erythroblasts, reticulocytes, and RBCs as a function of their CD44/FSC profiles. Representative CD44/FSC contour plots and quantifications in individual mice are presented (n=8-12 mice per group). **(C)** Spleen sizes in adult GULO^-/-^VitC^high^ and GULO^-/-^VitC^low^ mice were quantified as a function of their area (cm^2^) and means ± SEM (left) as well as representative images are shown (right). **(D)** Hematocrits in GULO^-/-^VitC^high^ and GULO^-/-^VitC^low^ mice were evaluated, and percentages are shown (n=5-6 mice with two replicates). Statistical analyses were performed using an unpaired two-tailed t-test in panels **(A, C, D)** and a two-way ANOVA (Tukey’s test) in panel **(B)**. *p < 0.05; **p < 0.01; ***p < 0.001; ns, not significant.

Unlike humans, mice exhibit erythropoietic activity in the spleen under stress conditions. It was therefore of interest to determine whether splenic erythropoietic activity is regulated by ascorbate levels. In accord with the data in 28 day old *Gulo^−/−^* VitC^low^ mice–showing decreased numbers of splenic Ter119^+^ cells (**Figure 5C**), erythroid cell numbers were also reduced at day 14 of life (p<0.05). However, Ter119^+^ cells were not impacted following ascorbic acid deprivation during the adult period (**Figure 6A**). To assess whether vitamin C deprivation resulted in a compensatory increase in splenic erythropoiesis or alternatively, a block in erythroid differentiation, we evaluated the relative levels of early and late erythroblasts in pups and adults. Erythropoiesis is maintained in the mouse spleen during the neonatal period, with erythroid precursors detected until 6-7 weeks of age (51–53) and indeed, we found that early erythroblasts, characterized as Ter119^+^CD44^hi^FSC^hi^, were detected in spleens of *Gulo^−/−^* VitC^high^ mice during the neonatal period but were almost undetectable in adults (20.4±3.6% *vs* 4.5±0.8%, **Figure 6B**). Importantly though, the percentages of early erythroblasts in both *Gulo^−/−^* VitC^low^ pups and adults was markedly increased as compared to ascorbate-replete animals (p<0.01, **Figure 6B**). The continued presence of early erythroblasts in *Gulo^−/−^* VitC^low^ adult mice (23.4±5.5%) reveals a secondary erythropoietic activity that was associated with a significantly increased spleen area in adult *Gulo^−/−^* mice (p<0.05, **Figure 6C**). It was though insufficient as the hematocrit in adult *Gulo^−/−^* mice was still reduced compared to ascorbate-sufficient mice (p<0.01, **Figure 6D**). Collectively, these data demonstrate the critical role of ascorbate metabolism in supporting splenic erythroid differentiation, with insufficient compensation in low ascorbate conditions.

### Anemia-induced erythropoiesis is dependent on ascorbic acid metabolism

**Figure 7:**
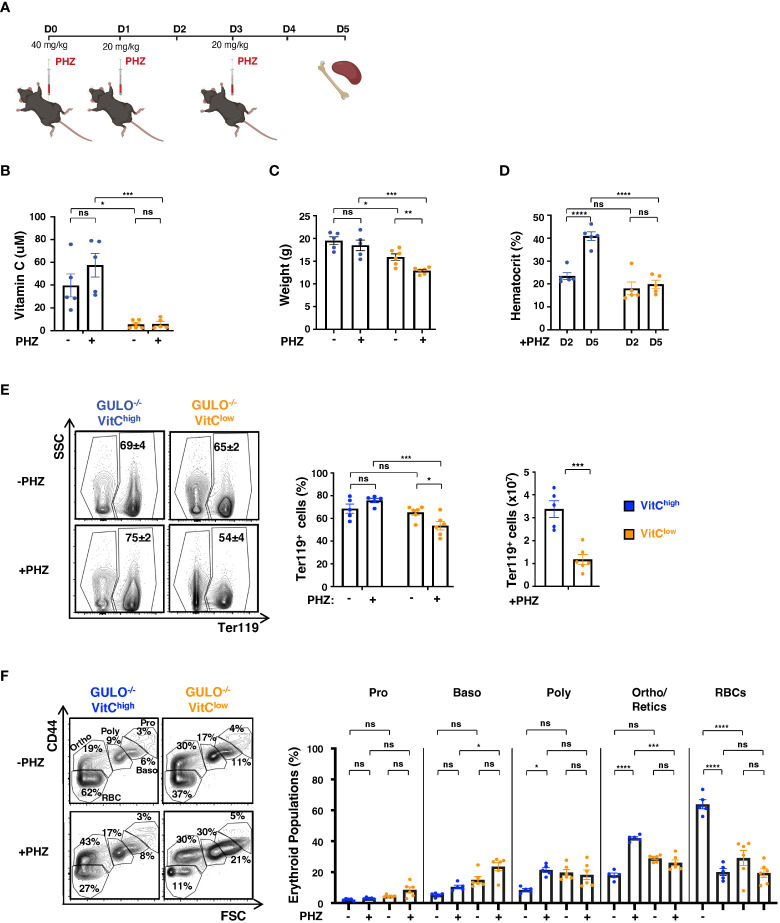
Hemolytic anemia results in changes in bone marrow erythroid differentiation in ascorbate-sufficient but not ascorbate-deficient GULO^-/-^ adults. **(A)** Schema illustrating the experimental protocol used to induce hemolytic anemia in adult GULO^-/-^ mice by phenylhydrazine (PHZ). PHZ was administered IP at a dose of 40mg/kg on day 0 and at 20mg/kg on days 1 and 3, and then mice were euthanized at day 5. **(B)** Plasma vitamin C concentrations were measured by HPLC in control and PHZ-treated GULO^-/-^VitC^high^ and GULO^-/-^VitC^low^ mice at day 5. Means ± SEM are presented (n=5-6 per group). **(C)** Weights of control and PHZ-treated GULO^-/-^VitC^high^ and GULO^-/-^VitC^low^ mice were measured at day 5 post-treatment. Means ± SEM are shown (n=5-6 per group). **(D)** Hematocrits were monitored in PHZ-treated GULO^-/-^ mice on days 2 and 5 and means ± SEM are presented (n=5 per group). **(E)** The impact of PHZ-induced hemolytic anemia on Ter119^+^ BM cells was evaluated at day 5 in the indicated conditions. Representative contour plots, percentages, and absolute numbers of Ter119^+^ cells are presented. **(F)** The differentiation state of Ter119^+^ erythroblasts was evaluated as a function of CD44/FSC profiles, distinguishing proerythroblasts (Pro), basophilic erythroblasts (Baso), polychromatic erythroblasts (Poly), orthochromatic erythroblasts (Ortho), reticulocytes, and RBCs as indicated (left). The percentages of cells in each stage of erythroid differentiation were quantified in the indicated conditions at day 5 of PHZ-induced anemia (right). Statistical analyses were performed using an unpaired two-tailed t-test in panels **(B–E)** and a two-way ANOVA (Tukey’s test) in panel **(F)**. *p < 0.05; **p < 0.01; ***p < 0.001; ****p < 0.0001; ns, not significant.

The data presented above demonstrated the importance of ascorbic acid for erythroid differentiation under physiological conditions and revealed the potential of splenic erythropoietic activity in adult *Gulo^−/−^* mice to compensate for ineffective erythropoiesis under conditions where ascorbic acid levels were limiting. It was therefore of interest to determine the role of ascorbic acid in responding to a pathological situation of hemolytic anemia. We therefore subjected adult *Gulo^−/−^* mice to a chemically induced hemolytic anemia by injecting phenylhydrazine (PHZ) over 5 days as shown in **Figure 7A**. Serum vitamin C levels were not altered by PHZ, remaining significantly reduced in the low-dose group (p<0.001, **Figure 7B**). While vitamin C deprivation was associated with a basal decrease in weight (34) (**Figure 7C**), PHZ treatment only impacted weight loss in this group (decrease of 15.1±0.3%, p<0.01, **Figure 7C**). Importantly, these *Gulo^−/−^* VitC^low^ mice did not exhibit an increase in hematocrit following PHZ-induced anemia. Hematocrits initially fell sharply in both groups at day 2, to 23.4±1.5% (*Gulo^−/−^* VitC^High^) and 18.1±2.8% (*Gulo^−/−^* VitC^Low^), as previously described (54). However, by day 5, the high dose vitamin C group recovered to 40.9±1.9% (p<0.0001) while the low dose group remained unchanged (**Figure 7D**). The percentage of Ter119^+^ BM erythroid cells was not increased following PHZ treatment of the vitamin C-high *Gulo^−/−^* mice group, but they were decreased in the vitamin C-low group, resulting in a 3-fold lower numbers of Ter119^+^ BM erythroid cells (p<0.001, **Figure 7E**). Furthermore, in the vitamin C-high *Gulo^−/−^* mice group, there were compensatory changes in erythroblast differentiation. Proerythroblasts, basophilic erythroblasts, polychromatic erythroblasts, orthochromatic erythroblasts/reticulocytes, and RBCs can be distinguished by their CD44/FSC profiles (55, 56) and we detected augmented BM polychromatic and orthochromatic erythroblasts following PHZ treatment (p<0.05-0.0001, **Figure 7F**). Importantly though, hemolytic anemia in vitamin C-low *Gulo^−/−^* mice did not result in significant changes in any of these BM erythroid subsets (**Figure 7F**).

**Figure 8:**
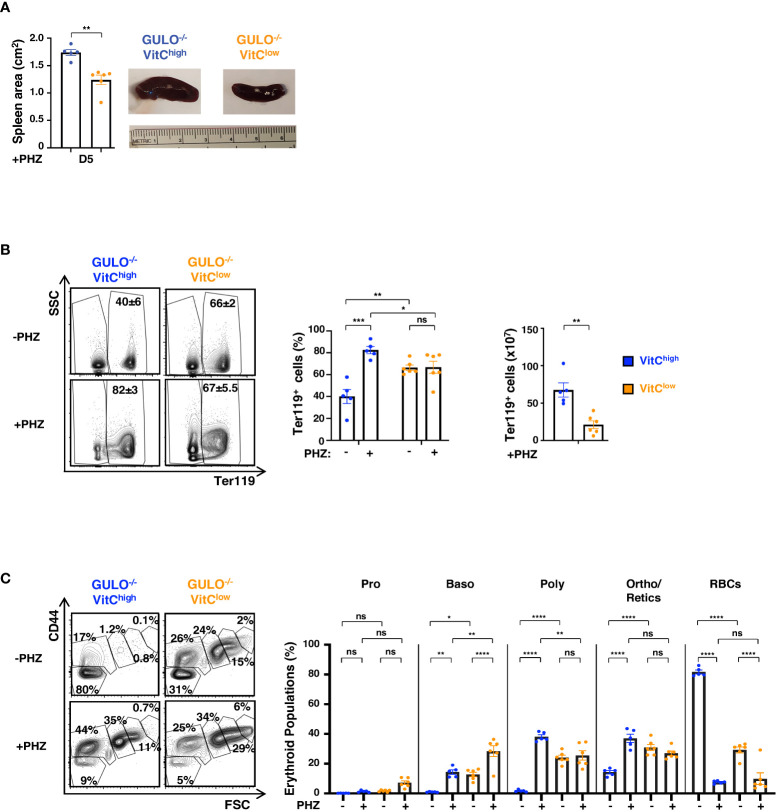
Impaired stress erythropoiesis in ascorbate-deficient GULO^-/-^ mice in response to acute hemolytic anemia. **(A)** Spleen area (cm^2^) in PHZ-treated GULO^-/-^ mice was measured at day 5 and means ± SEM (left) as well as representative images (right) are shown. **(B)** The impact of PHZ-induced hemolytic anemia on the percentages and absolute numbers of splenic Ter119^+^ cells in GULO^-/-^VitC^high^ and GULO^-/-^VitC^low^ mice are shown at day 5. Representative Ter119/SSC contour plots, percentages, and absolute numbers of Ter119^+^ cells are presented (n=5-6 mice per group). **(C)** Changes in splenic proerythroblast, basophilic, polychromatic, orthochromatic erythroblast, and reticulocyte subsets as well as RBCs were monitored in PHZ-treated GULO^-/-^VitC^high^ and GULO^-/-^VitC^low^ mice as described in (**Figure 7F**). Quantifications of erythroid precursors as well as representative CD44/FSC contour plots are shown. Statistical analyses were performed using an unpaired two-tailed t-test in panels **(A, B)** and a two-way ANOVA (Tukey’s test) in panel **(C)**. *p < 0.05; **p < 0.01; ***p < 0.001; ****p < 0.0001; ns, not significant.

As discussed above, mice generally respond to anemia by activating a stress erythropoiesis in the spleen (57–59). We therefore evaluated erythropoietic activity in the spleens of anemic *Gulo^−/−^* mice. An increased response of the vitamin C-high as compared to vitamin C-low group was demonstrated by the significantly increased spleen size in the former (p<0.01, **Figure 8A**). This was associated with a dramatic increase in the frequency of splenic Ter119^+^ erythroid cells in the vitamin C high group (p<0.001, **Figure 8B**). Most strikingly, vitamin C-high *Gulo^−/−^* mice responded to hemolytic anemia with increases in splenic basophilic, polychromatic, and orthochromatic erythroblast/reticulocyte subsets whereas the vitamin C-low group only exhibited increases in the basophilic erythroblast subset (**Figure 8C**). Thus, under conditions of hemolytic stress, vitamin C-deficient mice are not able to massively increase their erythropoietic activity, either in the BM nor spleen, resulting in an inability to overcome severe anemia.

## Discussion

Hematopoiesis is a dynamic process that allows all blood lineage cells to be maintained throughout life. This process relies on the continual presence of self-renewing, multipotent HSCs but it is notable that the perinatal period is a critical developmental window for immune cell and erythrocyte generation (60–62). Nonetheless, the differential roles of metabolic parameters in regulating perinatal versus adult hematopoiesis have not been extensively addressed. Using a murine GULO-deficient mouse model, we show that vitamin C deficiency during the perinatal but not adult period results in a hypocellular BM. These data suggested that perinatal hematopoiesis exhibits a significantly greater dependence on the vitamin C micronutrient than hematopoiesis occurring during the adult period and indeed, HSC, MPP, and HPC progenitors were dramatically reduced in BM of vitamin C-deficient *Gulo^-/-^* pups. Furthermore, our data show a differential impact of vitamin C on different hematopoietic lineages; neither BM nor splenic myeloid cells were reduced, even upon extended vitamin C deprivation, whereas B lymphoid and erythroid cells were markedly decreased under conditions of vitamin C deficiency. Thus, our data reveal a pivotal role for vitamin C in HSC maintenance during the postnatal period and point to differences in the sensitivity of different hematopoietic lineages to ascorbate metabolism.

One major difficulty in elucidating the mechanism(s) *via* which vitamin C exerts its actions on human hematopoietic differentiation is due to the lack of appropriate animal models. Aside from primates, who lost the ability to synthesize vitamin C at the split between the Strepsirrhini and Haplorrhini primate suborders (63, 64), guinea pigs and bats are the only two other species that have independently lost the ability to synthesize vitamin C due to mutations in the *Gulo* gene (20, 21, 65). As such, rat and murine models with mutations in the *Gulo* gene have been important for evaluations of physiological processes that are modulated by vitamin C levels (23, 66, 67). Notably though, these animal models are not flawless because they lack compensatory mechanisms that have evolved in higher primates. Additionally, while humans develop scurvy when plasma vitamin C levels are <10µM, mice can tolerate much lower plasma vitamin C levels (<1µM), highlighting important physiological differences between species (6, 68, 69).

Higher primates have likely been subjected to severe, but as yet unknown, environmental pressures that resulted in mutations in the *GULO* gene as well as in the urate oxidase (*UOX*) gene (70). In the 21^st^ century, the loss of the enzymes encoded by these genes increases the risk of *Homo Sapiens* to develop multiple diseases, including hypertension, cardiovascular disease, and infection, amongst others (70). That being said, humans appear to have evolved mechanisms to potentially overcome some of the drawbacks associated with significantly reduced levels of vitamin C. While the liver produces ascorbate at the rate of 200 mg/kg/day in vitamin C-producing mammals (71, 72), humans survive on massively lower levels, with FDA recommendation of 1-1.5 mg/kg/day (73). A further argument that strongly suggests that humans have evolved compensatory mechanisms for vitamin C loss is the finding that mice and rats with an inactivated *Gulo* gene require significantly higher doses of vitamin C than humans, ranging from 80-300 mg/kg/day (23, 66, 67). As fruit bats also consume more massive levels of vitamin C, reaching 250 mg/kg/day (74), it is tempting to hypothesize that they have not developed compensatory changes. Importantly, two known compensatory changes in humans are both associated with erythrocytes, the most abundant cell in the human body. First, human, but not mouse, erythrocytes contain an ascorbate-reducible b-type cytochrome, likely contributing to their ability to reduce the one electron-oxidized form of ascorbate, monodehydroascorbate. Moreover, a b-type cytochrome is also highly present on erythrocytes in guinea pigs, another mammal that lost the ability to produce vitamin C (75). Second, the postnatal erythroid expression of GLUT1, promoting the transport of glucose as well as dehydroascorbic acid (DHA)–the two-electron oxidized form of vitamin C (76–78), is a specific feature of those mammals that have lost the ability to synthesize ascorbate from glucose (57, 79). Indeed, human red blood cells harbor greater than 200,000 molecules of GLUT1 per cell (80, 81), allowing the shuttling of DHA across the membrane followed by its rapid reduction to ascorbate [reviewed by (82)]. Nonetheless, *Gulo^-/-^* mice with low ascorbate levels exhibit changes in multiple metabolites (83) and like humans, they develop vascular disease as demonstrated by aortic wall damage (23). Together, these data support the use of *Gulo^-/-^* mice to evaluate the impact of vitamin C deficiency on hematopoiesis.

The experiments presented here highlight the critical need for adequate serum vitamin C levels in hematopoiesis in the *Gulo^-/-^* mouse model, especially during the postnatal period. Previous research elegantly showed that vitamin C deprivation in adult mice results in an increased frequency of BM HSCs in the absence of any changes in the frequency of MPPs, HPCs, CMPs, or MEPs (10). In our studies, the marked BM hypocellularity in *Gulo^-/-^* pups, secondary to vitamin C deprivation in the lactating dams, was associated with a marked decrease in HSC, MPP, and HPC numbers but with no changes in the frequency of these cells. These data highlight differences in the impact of ascorbate depletion during the neonatal period as compared to the adult period. It should also be noted that ascorbate depletion was responsible for the loss of these progenitors during the neonatal period as progenitor numbers were rescued by a 2 week repletion with vitamin C. Our data also highlight differences in the dependence of more committed progenitors to ascorbate levels; while CMP and MEP cells were markedly reduced in *Gulo^-/-^* pups following vitamin C depletion in the dams, GMPs were maintained and their frequency was even augmented. This was associated with a maintenance of BM and splenic CD11b^+^ myeloid cells whereas Ter119^+^ erythroid cells were markedly reduced. While the mechanisms responsible for differential vitamin C dependence of GMPs and MEPs remains to be determined, it is important to note that vitamin C serves as a cofactor for the dioxygenase activity of Tet2 (84) and the effect of vitamin C on HSCs has been shown to be mediated, at least in part, by Tet2 (10, 11). Thus, it will be of much interest to evaluate how the kinetics of ascorbate depletion during the neonatal and adult periods regulates Tet2 activity in progenitor subsets. Our data strongly suggest that multiple hematopoietic processes, starting with the differentiation and maintenance of hematopoietic stem cells, are significantly more sensitive to vitamin C levels during the neonatal than the adult period.

As regards the impact of vitamin C deprivation on immune responsiveness, a large number of studies suggest that vitamin C supplementation may alleviate or prevent infections (4). Data emerging from the Covid pandemic has even pointed to a role for vitamin C in improving immune function in patients infected with SARS-CoV-2 (85, 86). Furthermore, a specific impact of vitamin C on immunity during the newborn period is suggested by an exciting recent study performed in the Central-African Republic; offspring born to vitamin C-deficient mothers had low serum vitamin C levels and they were at a significantly higher risk of being infected with an enteric virus (87). In our study, vitamin C deficiency resulted in a significant reduction in B lymphocytes in pups and an almost complete loss in the generation of B cells following 4 weeks of vitamin C deprivation. Furthermore, low numbers of peripheral B cells in *Gulo^-/-^* pups was associated with significantly reduced serum IgM/IgG levels. Taken together, it would be of much interest to determine whether humoral and cellular immunity are negatively impacted in infants, as well as adults, with hypovitaminosis-C.

Within the context of human erythrocyte differentiation, ascorbate has been found to play a role in red cell function. Serum ascorbate levels are inversely correlated with the osmotic fragility of red cells and moreover, in patients with diabetes, lower erythrocyte ascorbate levels are associated with increased RBC rigidity (69). Similarly, in patients with end-stage renal disease, low serum vitamin C levels correlate with hypochromic red cells whereas patients with high vitamin C levels harbor an increased fraction of red cells with normal hemoglobin content (88). These correlative studies are in agreement with *ex vivo* erythropoiesis assays showing that vitamin C promotes the erythroid differentiation of human hematopoietic progenitors (17). Furthermore, while there is a crosstalk between Tet2 and ascorbate, the mechanism is related to the protection of erythroblasts from oxidative stress (17, 89), in a manner that appears to be independent of Tet2’s dioxygenase activity (90, 91). The data presented here reveal the importance of serum ascorbate levels in supporting *in vivo* erythroid differentiation, with low levels of ascorbate severely attenuating the ability of vitamin C-deficient mice to respond to anemia.

Vitamin C supplementation has been evaluated as a means of improving erythroid differentiation in hemodialysis patients as well as in patients with anemia of chronic disease, and importantly, it has shown potential clinical benefit in some patients (92–95). However, the impact of vitamin C on erythropoiesis does not appear to be homogeneous; it did not improve hemoglobin recovery in patients with iron-deficiency anemia (96) and in patients with sickle cell disease (SCD, the combined treatment of vitamin C and vitamin E increased markers of hemolysis (97). Interestingly though, a study of SCD patients in Saudi Arabia revealed significantly lower plasma vitamin C levels compared to healthy controls (98). Together, these studies highlight the paucity of information regarding the mechanisms *via* which vitamin C regulates ineffective erythropoiesis. Our identification of an abnormal stress erythropoiesis in vitamin C-deficient *Gulo^-/-^* mice underscores the importance of developing new murine models for studying human erythroid pathologies, especially erythroid disorders that manifest during the newborn period.

## Methods and materials

### GULO^-/-^ Mice

GULO^-/-^ mice (23) were maintained and bred under specific pathogen-free conditions in the NIDDK animal facility (NIH, Bethesda, MD). Mice were fed *ad libitum* on a regular chow diet (NIH-07) on a 12h light/12h dark cycle and supplemented with ascorbic acid (Sigma-Aldrich, St. Louis, MO) in the drinking water. Water was changed every 2 days. In experiments performed on 14-17 day old GULO^-/-^ pups, dams were either maintained on 1g/L of ascorbic acid pre- and post-delivery (high vitamin C group) or started on 0.5g/l during pregnancy and then shifted to 0.1g/L post-delivery (low-vitamin C group, **Figure 1A**). For experiments performed on adult GULO^-/-^ mice (6-7 weeks of age), all mice were maintained on high vitamin C supplementation (1g/L in drinking water) until 14 days prior to experimentation when vitamin C levels were either maintained or decreased to 0.1g/L (**Figure 1B**). For rescue experiments, 14 day old GULO^-/-^ pups (and dams) in the low vitamin C group described above were shifted to high dose ascorbic acid (1g/L) for 14 days, until 28 days of age (referred to as VitC^low-rescue^, **Figure 2A**).

Animal care and experiments were performed in accordance with National Institutes of Health (NIH) guidelines and all experiments were approved by the Animal Care and Use Committee of the NIDDK.

### Induction of anemia

Haemolytic anaemia was induced in adult GULO^-/-^ mice (**Figure 5A**) maintained on either high (1g/L) or low (0.1g/L) vitamin C supplementation. Anemia was induced by phenylhydrazine hydrochloride (PHZ, Sigma-Aldrich) diluted in PBS. Mice were injected intraperitoneally at days 0, 1, and 3, with doses of 40mg/kg, 20mg/kg, and 20mg/kg, respectively. Haematocrits were measured in retroorbital samples on days 2 and 5. Mice were euthanized on day 5 for analyses.

### Vitamin C and haematocrit measurements

Whole blood was collected by retro-orbital bleeds using heparinized capillary tubes and haematocrits were evaluated. For vitamin C measurements, plasma was further processed for HPLC analyses as described previously (99). Briefly, whole blood was centrifuged at 250g for 5 min at 4C. One volume of plasma was mixed with four volumes of cold methanol/1mM EDTA and centrifuged at >14000g for 10 min at 4C. The supernatant was then transferred to a fresh tube and either analyzed by HPLC or stored at -80C. Ascorbic acid was analyzed by HPLC coupled with electrochemical detection (100).

### Thymocyte, splenocyte and BM preparations

Murine thymus, spleen, and bone marrow were collected after euthanizing the mice. BM was flushed using a 25gauge needle into 2% fetal bovine serum (FBS) PBS. Single cell thymocyte and splenocyte suspensions were generated by physical disruption of tissue and filtration through 70µm nylon cell strainers. Cells were counted and further processed for flow cytometry.

### Flow cytometry

Single cell suspensions were stained with three separate antibody cocktails for analyses of BM, spleen, and thymus. BM subsets were identified using the following conjugated mAbs: Ter119, CD25 (Invitrogen, Thermo Fisher Scientific, Waltham, MA), c-kit, Sca1, CD19 and CD44 (BD Biosciences, San Jose, CA). Lineage-differentiated BM cells were eliminated using a dump consisting of mAbs against CD11b, Gr1 and CD3. HSCs, MPPs, and HPCs were identified within the lineage^-^ Sca1^+^c-kit^+^ (LSK) lineage as a function of CD150 and CD48 profiles while CMP, GMP, and MEP were evaluated on lineage^-^c-kit^+^ (LK) progenitors as a function of CD34 and CD16/32 expression (36, 38). For thymus, cells were stained with the following directly conjugated mAbs: CD3, c-kit, CD4, CD8, CD44, TCRγδ (BD Biosciences) and CD25 (Invitrogen). Non-T lineage cells were excluded using a dump consisting of mAbs against CD19, Gr1, CD11b. Splenic subsets were distinguished using the following directly conjugated mAbs: Ter119 (Invitrogen), CD44, CD3, CD4, CD8, CD62L, TCRγδ, CD19 and CD11b (BD Biosciences). Stained cells were analyzed by flow cytometry using the LSR II-Fortessa (BD Biosciences). All data analyses were performed using Diva (BD Biosciences), and FlowJo Mac v.10.6.2 software (Tree Star).

### ELISA

The total serum IgM, IgG and IgA protein levels were measured by ELISA (Thermo-Fisher Scientific). Plates were coated with respective purified and pre-titrated monoclonal antibodies and developed with pre-titrated HRP-conjugated anti-mouse polyclonal antibodies specific for each mouse Ig isotype. Isotype controls were serially diluted and used to generate a standard curve. The serum samples were diluted, added to the plate as technical triplicates and their Ig concentrations were determined using the SoftMax-Pro software (Molecular Devices, LLC, San Jose, CA).

### Statistical analyses

Data were analysed using GraphPad Prism software version 9.3.1 (Graph Pad Prism, La Jolla, CA). p-values were calculated using either unpaired two-tailed t-tests, one-way ANOVA (Tukey’s multiple comparison test) or two-way ANOVA (Tukey’s multiple comparison test), as specified. p-values are indicated in individual figure legends.

## Data availability statement

The raw data supporting the conclusions of this article will be made available by the authors, without undue reservation.

## Ethics statement

This study was reviewed and approved by the Animal Care Committee of the NIDDK, National Institutes of Health (NIH).

## Author contributions

IP, MP, AM, VZ, ML, PCV, and NT conceived the study. IP, MP, AM, JM, PGM, SK, VZ, ML, PCV, and NT were involved in study design. IP, MP, AM, JM, VZ, and PCV performed experiments. VZ, ML, PCV, and NT supervised the study. All authors participated in data analysis and discussions. IP, MP, PCV, and NT wrote the manuscript and all authors critically reviewed the manuscript. All authors contributed to the article and approved the submitted version.

## Funding

IP, AM, and PGM were funded by fellowships from the French Labex EpiGenMed, the French Ministry of Health, and the Clarin-COFUND EU Program. IP and AM were also funded by the intramural NIH research program and PGM by NIH DK32094. This work was supported by generous funding from the FRM, ARC, the French national (ANR) research grants and the French laboratory consortiums (Labex) EpiGenMed and GR-EX. ML and PCV are supported by the NIDDK (ZIA DK054506 23) and NT by NCI intramural NIH research programs (ZIA BC 011924 and ZIA BC 011923).

## Acknowledgments

We thank all members of our lab for their scientific critique and support and are grateful to the NIH animal facility staff for their expert assistance. The schematics were created with BioRender.com.

## Conflict of interest

The authors declare that the research was conducted in the absence of any commercial or financial relationships that could be construed as a potential conflict of interest.

## Publisher’s note

All claims expressed in this article are solely those of the authors and do not necessarily represent those of their affiliated organizations, or those of the publisher, the editors and the reviewers. Any product that may be evaluated in this article, or claim that may be made by its manufacturer, is not guaranteed or endorsed by the publisher.
