# Supplementary material for: Vitamin C deficiency reveals developmental differences between neonatal and adult hematopoiesis
Source: Front Immunol. 2022 Sep 30;13:898827. doi: 10.3389/fimmu.2022.898827 (PMC9562198; doi:10.3389/fimmu.2022.898827)
Supplement: Supplementary file 1 [file DataSheet_1.pdf]

Supplementary Figures

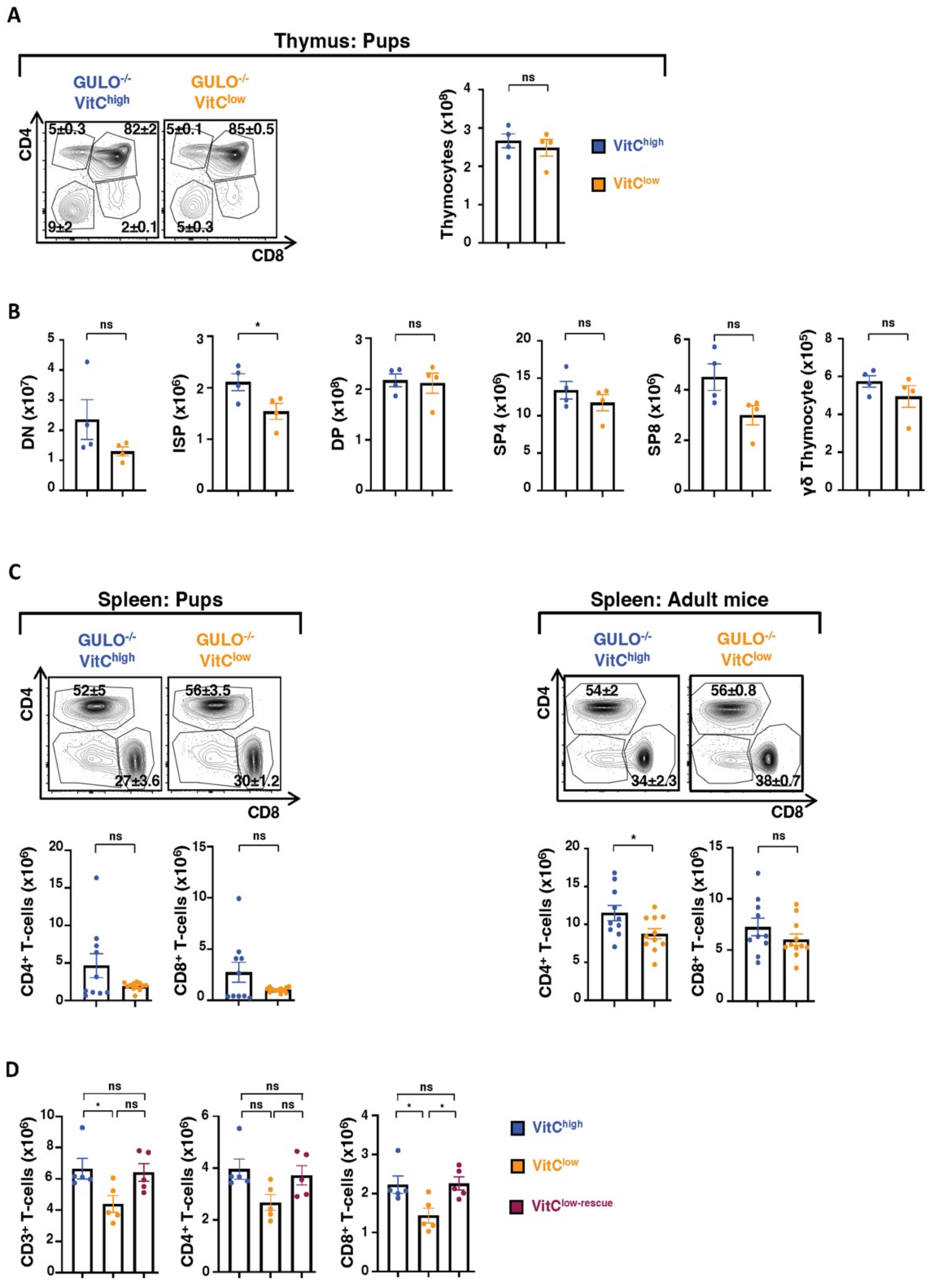

Figure S1. T cell differentiation is maintained following short-term ascorbate withdrawal in GULO$^{-/-}$ pups and adult mice.

(**A**) Representative CD4/CD8 thymus profiles from 14-day old GULO$^{-/-}$VitC^{high} and GULO$^{-/-}$VitC^{low} mice are shown (left). Total thymocyte numbers were quantified and means ± SEM are presented (right, n=4 mice per group). (**B**) CD4^{-}CD8^{-}CD3^{-} double negative (DN), CD4^{-}CD8^{+}CD3^{-} immature single positive (ISP), CD4^{+}CD8^{+} double positive (DP), CD4^{+}CD3^{+} single positive (SP4), CD8^{+}CD3^{+} single positive (SP8) and $\gamma\delta$ thymocytes were quantified in thymi from 14-day old GULO$^{-/-}$VitC^{high} and GULO$^{-/-}$VitC^{low} mice and means ± SEM are presented. (**C**) Representative CD4/CD8 contour plots within the CD3^{+} gate are shown (top) and CD4^{+} and CD8^{+} T cell numbers were quantified in spleens of pups (left) and adult GULO$^{-/-}$VitC^{high} and GULO$^{-/-}$VitC^{low} mice (bottom). (**D**) CD3^{+} T cells, as well as CD4+ and CD8+ subsets, were quantified in spleens of 28 day old GULO$^{-/-}$ mice maintained under VitC-high, VitC-low, and rescue conditions (n=5 mice per group). Means ± SEM are presented. Statistical analyses were performed using an unpaired two-tailed t-test for panels A-C and a one-way ANOVA (Tukey's test) for panel D. *p<0.05; ns, not significant

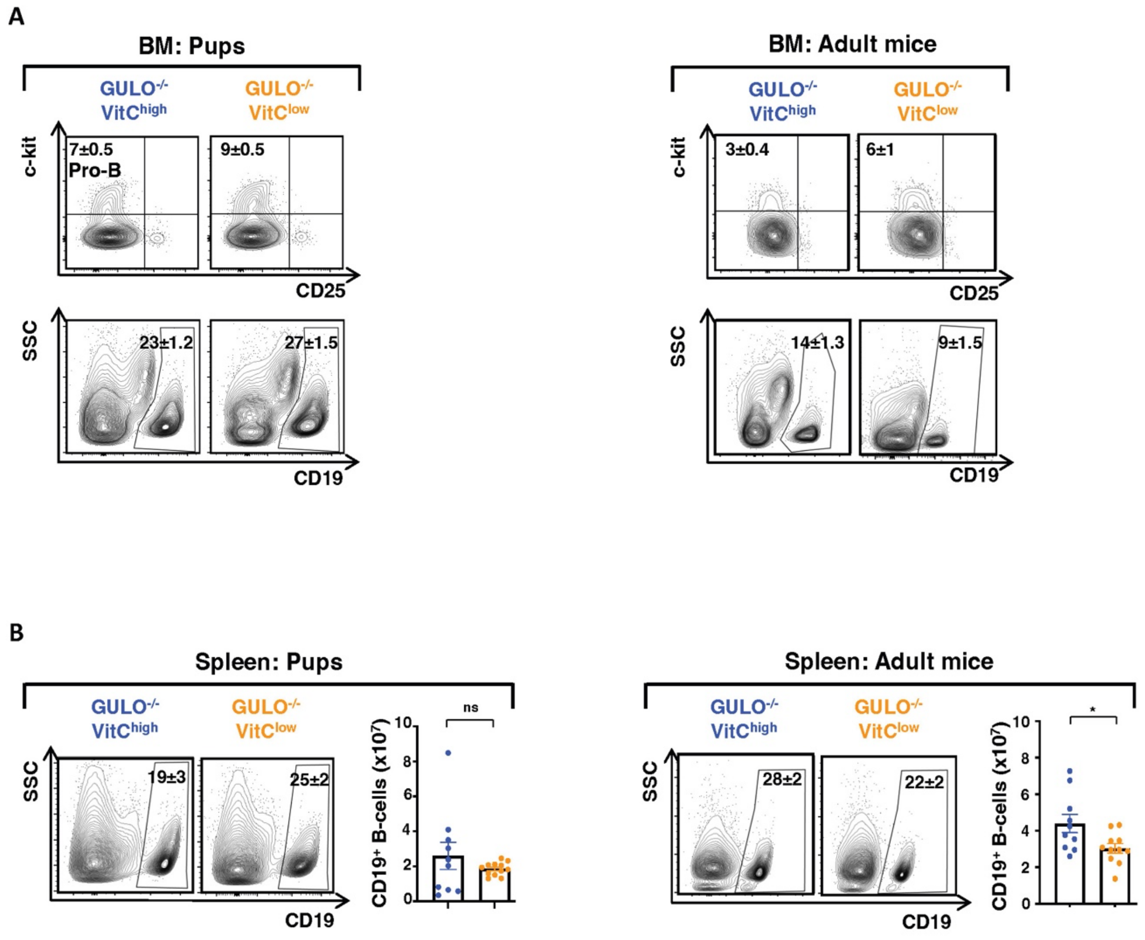

Figure S2. Splenic B cells are maintained in GULO$^{-/-}$ pups.
(**A**) Representative contour plots of c-kit^{+}CD19^{+} Pro-B and CD19^{+} BM B cells in pups (left) and adult (right) GULO$^{-/-}$ mice corresponding to data presented in Figure 4A. (**B**) The numbers of splenic CD19^{+} B cells in the indicated groups are presented as means ± SEM and representative contour plots are shown (n=10-12 mice per group). Statistical analyses were performed using an unpaired two-tailed t-test. *p<0.05; ns, not significant
